# *Lantana camara* plant extract catalyzed biosynthesis of graphene-metal nanocomposites with potential cytotoxic activities

**DOI:** 10.1371/journal.pone.0314850

**Published:** 2025-03-12

**Authors:** Kfait Ullah Khan, Misbahur Rehman, Mariam Hameed, Khyzar Hayat, Farhan Saeed, Muhammad Afzaal, Noor Akram, Faiyaz Ahmed, Aasma Asghar, Gebremichael Gebremedhin Hailu

**Affiliations:** 1 Department of Chemistry, University of Lahore, Sargodha Campus, Punjab, Pakistan; 2 Department of Food Science, Government College University Faisalabad, Faisalabad, Pakistan; 3 Food Safety and Biotechnology Lab, Department of Food Sciences, Government College University Faisalabad, Faisalabad, Pakistan; 4 Department of Basic Health Sciences, College of Applied Medical Sciences, Qassim University, Buraydah, Saudi Arabia; 5 Department of Nutritional Science, Government College University Faisalabad, Faisalabad, Pakistan; 6 Food Technology and Process Engineering, Oda Bultum University, Chiro, Ethiopia; University of Sharjah, UNITED ARAB EMIRATES

## Abstract

This study investigates the synthesis and characterization of Plant-Ag-graphene nanocomposites through a combination of spectroscopic and microscopic techniques, the nanocomposites were formed by catalyzing silver nanoparticles with plant extracts, and the resulting structures were analyzed using advanced instrumentation. In the FTIR analysis, distinctive peaks were observed at 3340 cm⁻^1^ (O-H stretching), 1740 cm⁻^1^ (C = O stretching), and 1050 cm⁻^1^. When compared to silver nanoparticles, the nanocomposites exhibited altered peak intensities, indicating modifications in chemical bonding. SEM images revealed that in nanocomposites, nanoparticles were adhered to graphene sheets, confirming the successful formation of Plant-Ag-graphene structures. EDX spectra showed a reduction in the silver content, confirming the integration of graphene into the nanocomposites. XRD analysis confirmed the presence of face-centered cubic-shaped Ag metal in the nanocomposites, while graphene exhibited a hexagonal crystalline shape. UV-Vis spectroscopy demonstrated shifts in peak positions, Spectrum A (400 to 700 nm) and spectrum B (265 nm), suggesting the successful synthesis of Plant-Ag-graphene nanocomposites. Moreover, the cytotoxic activity showed cell inhibition among Plant-Ag-Graphene (65.69%) and Plant-Ag (61.39%), respectively.

## 1. Introduction

Nanoparticle-based medications were established via a lot of hard efforts. They use nanostructured materials created by combining bioactive chemicals with inorganic nanostructured matrix. Metal nanoparticles have been widely employed in a variety of fields, including antibiotic, antiviral, diagnostics, antitumor, and targeted drug delivery due to their unique physiochemical properties [1]. They may combine biopolymers that imitate the organic components of the matrix that surrounds cells of bone with bioactive nanoceramics to promote bio mineralization [2]. Nanoparticles antimicrobial action is either direct (destroying/penetrating the cell envelope, oxidizing cytoskeleton, or interfering with trans-membrane electron transfer) or indirect (producing byproducts) [3]. Because of its inexpensive price, ease of use, and strong resistance to photo-induced disintegration, titanium oxide is amongst the most investigated semiconductors for photo-catalytic processes [4]. These metal nanoparticles have excellent performance and durability in addition to being inexpensive and safe for consumption. Owing to these qualities, the American FDA has approved its utilization in food for humans, medicines, skincare, and contact with food products [5]. Each carbon atom in graphene forms strong covalent bonds with three neighboring carbon atoms, resulting in a robust yet lightweight structure. This arrangement allows graphene to withstand considerable mechanical stress and deformation, making it one of the strongest materials known to humanity. Its flexibility enables it to bend and stretch without losing its structural integrity [6,7].

The exceptional electrical, mechanical, thermal and chemical characteristics of graphene have piqued the scientific community curiosity (Jariwala et al., 2013). It is practically transparent, has a lower density than steel and transmits heat and electricity efficiently [8]. Moreover, metal nanoparticle attachment to graphene aids in the dry condition suppression of agglomeration of the resultant graphene sheets, although, increasing the distance amid the graphene layers and serving as a spacer, the metal nanoparticles enable approach to both sides of the graphene layer [9]. Currently, graphene-metal-nanocomposites have been used in therapeutic applications as photothermal therapy, biosensing, and bioimaging, drug and gene delivery and tissue engineering of stem cell. Reduction, hydrothermal, electrochemical, and ex-situ processes have all been used to create graphene/nanoparticle hybrid materials [10]. A recent study discovered a brand-new, simple, affordable, ecologically friendly, and scaleable method for creating few-layer graphene flakes that makes use of mild sonication and environmentally safe plant extracts [11]. Natural products have gained great interest in the drug discovery and development process due to their unique structures and a broad spectrum of biological effects [12].

About 150 different herb species belong to the genus *Lantana* which grows beneath bushes and shrubs that are between 0.5 and 2 meters tall. According to Linnaeus description in Species Plantarum from 1753, the genus *Lantana* has seven species six of which can be found in South America and one in Ethiopia, however, a few species also present in tropical Asia and Africa, *Lantana* is mostly found in tropical and subtropical America. Allopathic is a phenomenon in which alien plants produce chemicals that prevent native plants from growing. It shows the interrelationships between plants that are a part of chemical biodiversity [13,14]. *Lantana* is a crucial plant to study phytochemistry in since some species of the plant contain dangerous compounds. The major active principle of *Lantana camara*, Lantanin, with the molecular formula C_32_H_44_O_5_, was first systematically studied by Louw in 1943. After Lantadene B, with the chemical formula C33H4805, was extracted from all parts of the *Lantana* shrub, he called it Lantadene A in 1948 [15]. Nearly every bioactivity of *Lantana*, even those that are antipyretic, antibacterial, anti-mutagenic, antiseptic, anti-fungal, antiparasitic, and nematicidal, is attributed to lantadenes. Numerous of these biological effects may be attributed to the bioactive components of *Lantana*, including its alkaloids, terpenoids, phenolics, iridoid glycosides, furanonaphthoquinones, flavonoids, and phenyl ethanoid glycosides [16], however, the extracts from the foliage exhibit nematicidal, antifungal, antibacterial, and antiparasitic effects [17].

Various studies have been performed on development pf silver nanoparticles via suing natural bioactive compounds from plant extracts, as, *Salvia* species have long been utilized in medicinal and food sectors owing to their abundant secondary metabolites like flavonoids and phenolic compounds. In a research endeavor, *Salvia aethiopis L.* underwent a two-hour heating process in distilled water. Following filtration, the resulting water extract was subjected to treatment with silver nitrate to produce silver nanoparticles (Sa-AgNPs) [18]. Additionally, another recent study conducted green synthesis of silver nanoparticles using *Echinacea purpurea (L.)* Moench. Both the resulting AgNPs and the extract demonstrated remarkable antioxidant activity, suggesting potential applications in the food and pharmaceutical industries [19]. In another investigation, silver nanoparticles (AgNPs) were synthesized utilizing oleuropein to evaluate their antioxidant potential. The utilization of natural bioactive compounds in the biosynthesis of nanoparticles exhibited greater inhibitory effects [20]. Silver nanoparticles were also biosynthesized through the utilization of *Origanum majorana L.*, followed by the assessment of their antioxidant activity. Both the AgNPs and the extract displayed outstanding antioxidant activity, suggesting potential applications in the food and pharmaceutical sectors [21]. The body of literature presented supports the growing interest in utilizing natural bioactive compounds from plant extracts for the synthesis of silver nanoparticles (AgNPs). Therefore, in the present study, Plant-Ag-graphene nanocomposites were biosynthesized using plant extract of *Lantana camara.* Later on, cytotoxic potential was assessed of developed nanocomposites.

## 2. Materials and methods

### 2.1. Materials

**Fig 1:**
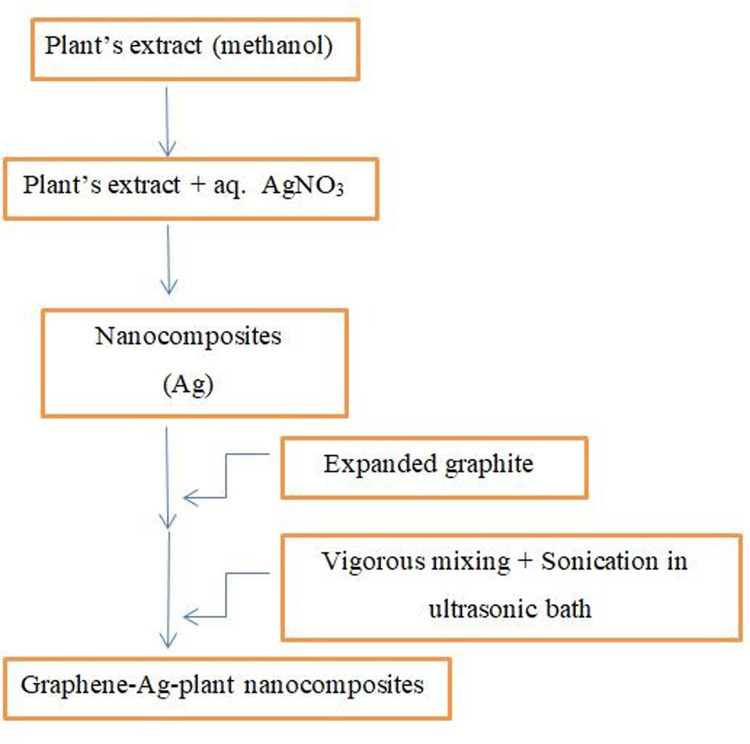
Experimental design.

*Lantana Camara* plant was collected from the Hilly valley of Musa Khel Mianwali District of Punjab Pakistan and dried in a shade to avoid exposure to direct solar light. It was grinded in common house hold grinder packed in Polythene bag and placed for further use. Chemicals and reagents used in this study were sourced from Sigma-Merck (Germany). This article does not contain any studies with human participants or animals performed by any of the authors. The experimental design had been showed in Fig 1.

### 2.2. Extraction of plant extract

Powder was made of *Lantana camara* leaves using a grinder, the powdered *Lantana camara* leaves (5 g) were added to lab graded Methanol (100 mL), the mixture was heated at 40 °C for 2 hours, after 2 hours, the mixture was filtered using Whatman filter paper to obtain a clear extract solution [22].

### 2.3. Preparation of plant catalyzed silver nanoparticles

In a solution comprising 0.017 grams of silver nitrate dissolved in 100 milliliters of distilled water, the mixture was agitated and maintained at a temperature range between 90 °C to 100 °C for an hour. Subsequently, 10 milliliters of *Lantana camara* plant extract were introduced into 90 milliliters of the prepared AgNO3 solution. The resulting admixture underwent vigorous stirring at 100 °C for a duration of one hour. A discernible alteration in color was observed, transitioning progressively from green to a pale green hue, eventually culminating in a brown coloration. The complete manifestation of this color change served as an indicative marker for nanoparticle formation, commencing within 10 to 15 minutes and reaching completion between 45 to 60 minutes. The resultant nanoparticles were subjected to purification through centrifugation at 15,000 revolutions per minute for 20 minutes, followed by their suspension in deionized water. Subsequently, the suspension was left to undergo drying, yielding a dried powder suitable for various analytical assessments [18].

### 2.4. Preparation of graphene-metal nanocomposites

20 milliliters of sulfuric acid (H2SO4) were mixed with 1 gram of expanded graphite under agitation at room temperature for 45 minutes. Subsequently, 2 grams of potassium permanganate (KMnO4) were added, and the mixture was swirled for an additional hour. Upon complete dissolution, the solution began to exhibit a gel-like consistency, prompting its transfer to a water bath at 80 °C for 5 minutes. The resulting slightly gelatinous material was subjected to vacuum filtration, with the filtrate subsequently dried and washed with distilled water until achieving neutralization. The dried material was then further dried in an oven at temperatures up to 70 °C [23].

### 2.5. Preparation of graphene-ag-plant nanocomposites

In a volume of 100 milliliters, plant-catalyzed silver oxide nanocomposites were prepared, into which 1 gram of expanded graphite and 10 ml of LC extract was introduced under stirring. The resulting mixture underwent sonication for a duration of one day at 60 °C. Simultaneously, expanded graphite was incorporated into 100 milliliters of plant extract, and the resultant blend underwent sonication for a day at 60 °C following the method outlined by Salunke and Kim [11]., to facilitate graphene production. Control over the sonication bath temperature was achieved by modulating the water content and covering the apparatus. Following synthesis, the produced nanocomposites were allowed to dry and subsequently stored in sample vials for subsequent analysis.

### 2.6. Characterization of nanocomposites

Synthesized Silver nanocomposites and Graphene-Silver-Plant nanocomposites were characterized in terms of Morphological and Molecular characterization. Thermo-Nicolet FTIR Spectrophotometer was used for molecular characterization available at University of Agriculture Faisalabad. The scanning electron microscope, SEM (Emcraft cubeseries, South Korea) available at department of physics Government College University Faisalabad was used for the morphological characterization. Moreover, SEM equipped with an EDX detector was used to perform Energy-Dispersive X-ray Spectroscopy. X-ray Diffraction (XRD) was performed over X-ray Diffractometer. However, UV-Visible spectroscopy, conducted using the UV-1280 Spectrophotometer.

#### 2.6.1. Cytotoxic activities.

The effects of the synthesized derivatives, encompassing *Lantana camara* leaf extract, Graphene-Ag-*Lantana camara* nanocomposites, and *Lantana camara*-silver nanocomposites, were assessed against the Huh7 human liver tumor cell line. Viability of the cells was evaluated using the Sulforhodamine B (SRB) assay. Cell cultivation was conducted at a constant temperature of 37 °C. For each experimental trial, cells treated with dimethyl sulfoxide (DMSO) were employed as the control group. Subsequently, the cells were exposed to a concentration of 500 micrograms per milliliter (µg/mL) of the Sulforhodamine B reagent, followed by an incubation period of 48 hours. The outcomes were quantified as a percentage of viable cells, offering insights into the cytotoxic effects of the synthesized derivatives on the Huh7 liver tumor cell line [24].

### 2.7. Statistical analysis

Additionally, cytotoxic activity data were statistically analyzed with XLSTART; the mean, standard deviation through one-way analysis of variance (ANOVA). Three replicates of each analysis were performed, and differences were deemed statistically significant at p ≤ 0.05.

## 3. Results and discussions

### 3.1. Infrared spectroscopy

**Fig 2:**
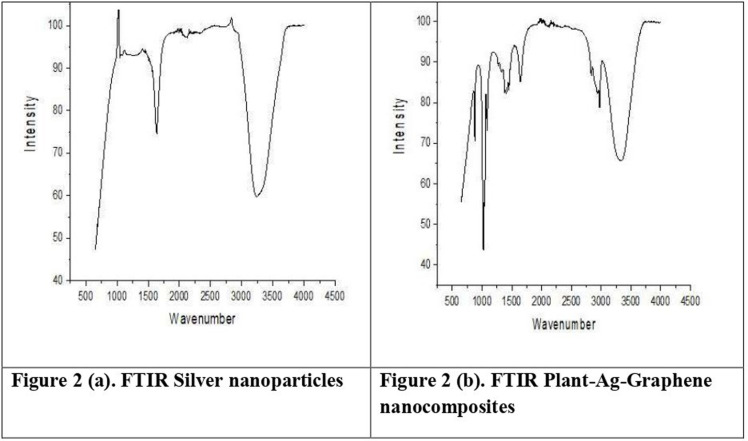
(a) FTIR Silver nanoparticles. (b) FTIR Plant-Ag-Graphene nanocomposites.

In the spectrum as showed in Fig 2a and b of silver nanoparticles, characteristic peaks are observed at approximately 3340 cm^-1, corresponding to O-H stretching vibrations, while the peak attributed to C = O stretching vibrations appears around 1740 cm^-1. Additionally, a minor peak is discernible near 1050 cm^-1. Conversely, in the spectrum of Graphene-Ag-plant nanocomposites, the intensity of the N-H peak appears diminished, and a peak around 3500 cm^-1 displays reduced intensity compared to the silver nanoparticles. Similarly, the peak associated with C = O stretching vibrations at approximately 1740 cm^-1 exhibits decreased intensity and a slight blue-shift relative to the silver nanoparticle spectrum. Notably, the peak observed around 1100 cm^-1 manifests heightened intensity compared to the Plant-Ag nanoparticles peak. These observations suggest distinct spectral characteristics indicative of the structural composition and interactions within the Graphene-Ag-plant nanocomposites. Results if the current study have been aligning with the findings of Wen et al. [22], who had developed graphene and metal nanoparticles using *Xanthium strumarium* plant extract, the FTIR spectroscopy had showed strong molecular bonding between graphene/silver, graphene/gold and plant extract. Similar findings have also been observed among the work of Ajitha et al. [25], who had developed silver nanoparticles and the FTIR analysis evidences the presence of various functional groups of biomolecules of LE is responsible for stabilization of AgNPs.

### 3.2. Scanning electronic microscopy (SEM)

**Fig 3:**
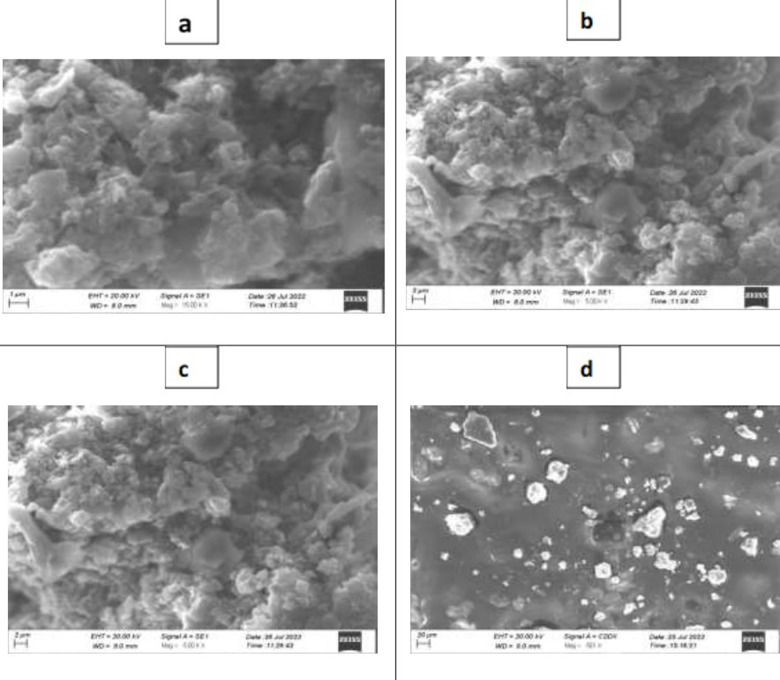
(a–d) Scanning electron microscopy of *Lantana camara*-Ag-graphene nanocomposites.

SEM (scanning electron microscopy) images in Fig 3a–d provide visual evidence of these phenomena, offering insights into the morphology and structure of the nanocomposite material at different length scales. The ability to visualize nanoparticle clustering and their interaction with the substrate is crucial for understanding the underlying mechanisms governing the properties of these materials.

These results suggest that the nanocomposites being studied exhibit a range of sizes in terms of nanoparticle distribution. Some nanocomposites show nanoparticle clusters that can be observed at scales up to 200 nanometers, while others display larger clusters extending up to 1 micrometer. The most detailed images reveal nanoparticle clustering on the graphite sheet, with visibility extending up to 20 micrometers.

This variation in scale implies differences in the distribution and aggregation of nanoparticles within the nanocomposite material. The observation of nanoparticles adhering to the graphite sheet rather than existing as isolated entities suggests a strong interaction between the nanoparticles and the graphite substrate. This interaction can potentially influence the properties and behavior of the nanocomposite material, such as its conductivity, mechanical strength, or catalytic activity, depending on the specific application. The results of the current study have been aligned with the finding of Ajitha et al. [25], who observed that the formation of spherical shaped nanoparticles and as AgNO3 concentration is increased, there was an increment in the particle size.

### 3.3. E nergy-dispersive X-ray spectroscopy (EDX)

**Fig 4:**
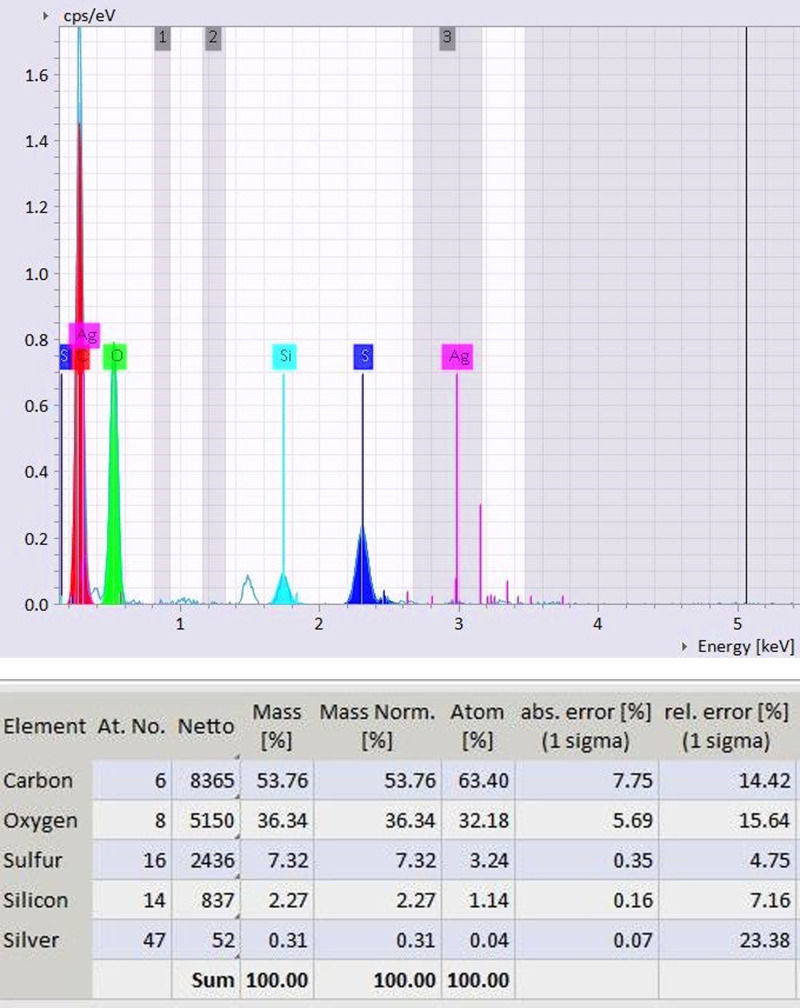
(a) EDX Spectra of *Lantana*-Ag-graphene nanocomposites. (b) EDX element concentration of *Lantana*-Ag-graphene nanocomposites.

The spectral analysis as showed in Fig 4a and b reveals distinct peaks corresponding to silver and graphene components, as well as peaks indicative of carbon, sulfur, oxygen, silicon, sodium, and calcium within the nanocomposite material. Notably, the silver peak in the spectrum demonstrates the formation of plant-graphene-silver nanocomposites.

Comparing the elemental composition obtained from energy-dispersive X-ray spectroscopy (EDX) of the silver nanoparticles previously reported, a notable discrepancy arises. While the reported EDX analysis of silver nanoparticles displayed a predominant silver content of 61%, the silver content within the nanocomposite spectra registers at 28%. Furthermore, the absence of carbon peaks in the reported EDX analysis of silver nanoparticles, juxtaposed with their presence in the current nanocomposite spectra, provides compelling evidence for the synthesis of graphene-silver nanocomposites.

This discrepancy in silver content, along with the presence of carbon peaks, underscores the distinct compositional nature of the synthesized nanocomposite material. Such spectral analysis not only confirms the formation of graphene-silver nanocomposites but also sheds light on the nuanced elemental distribution within the composite structure. These findings hold significance in elucidating the fabrication process and characterizing the properties of the resulting nanocomposite material, offering valuable insights for various scientific and technological applications. Results were in line with the findings of Mahadevaswamy et al. [26], who developed nickel oxide nanoparticles (NiO NPs) using *Lantana camara* flower extracts, EDX reveals the presence and formation of Ni, O, and C as the main elemental composition along with the traces of other elements such as Si, P, K, and Cl that are formed during the formation of NiO NPs present in the *L. camara* flower extract.

### 3.4. X-ray diffraction

**Fig 5:**
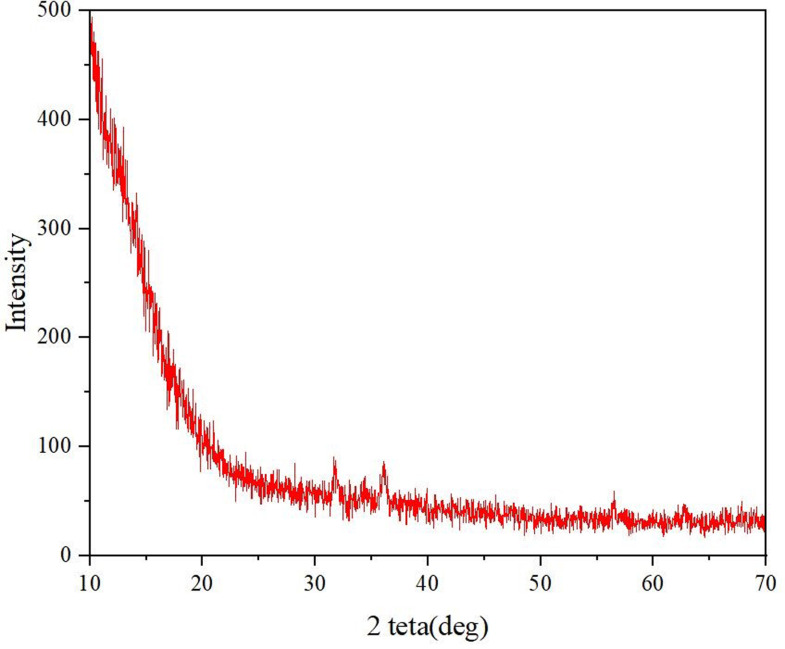
XRD spectra of *Lantana camara*-Ag-graphene nanocomposites.

X-ray diffraction (XRD) analysis as showed in Fig 5, yielded a distinct spectrum, revealing notable peaks near angles 33, 35, and 57 with intensities nearing 100, indicative of the presence of metallic silver (Ag). These prominent peaks affirm the crystalline nature of the Ag metal within the nanocomposite structure. Specifically, the presence of peaks at these angles suggests the face-centered cubic (FCC) crystallographic arrangement characteristic of silver. Concurrently, the XRD spectrum also exhibits a discernible peak occurring at 2θ =  26.49°, corresponding to the graphene component of the nanocomposites. This peak signifies the hexagonal crystalline structure inherent to graphene. The coexistence of peaks representing both Ag and graphene crystalline phases further elucidates the composite nature of the material under examination.

The observed crystallographic features, characterized by the FCC shape of silver and the hexagonal shape of graphene, provide crucial insights into the structural properties of the nanocomposites. Such findings not only confirm the presence of both constituents but also underscore the potential for tailored properties arising from the synergistic interaction between the metallic and carbonaceous components. This detailed structural analysis serves as a fundamental step towards understanding the crystalline characteristics and overall behavior of the nanocomposite material, facilitating targeted applications in diverse fields such as catalysis, sensing, and electronics. Results have been aligned with the findings of Govindasamy et al. [27], the XRD analysis confirms the formation of a single-phase orthorhombic Y_2_O_3_ structure, revealing the successful synthesis of Y_2_O_3_ NPs. Results were also in line with the findings of Kumar et al. [28], who had developed silver nanoparticles (AgNPs) using *Lantana camara L.* flower extract, and observed the formation of pure silver metal with face-centered cubic symmetry and confirms crystalline nature among XRD analysis.

### 3.5. Ultraviolet visible spectroscopy

**Fig 6:**
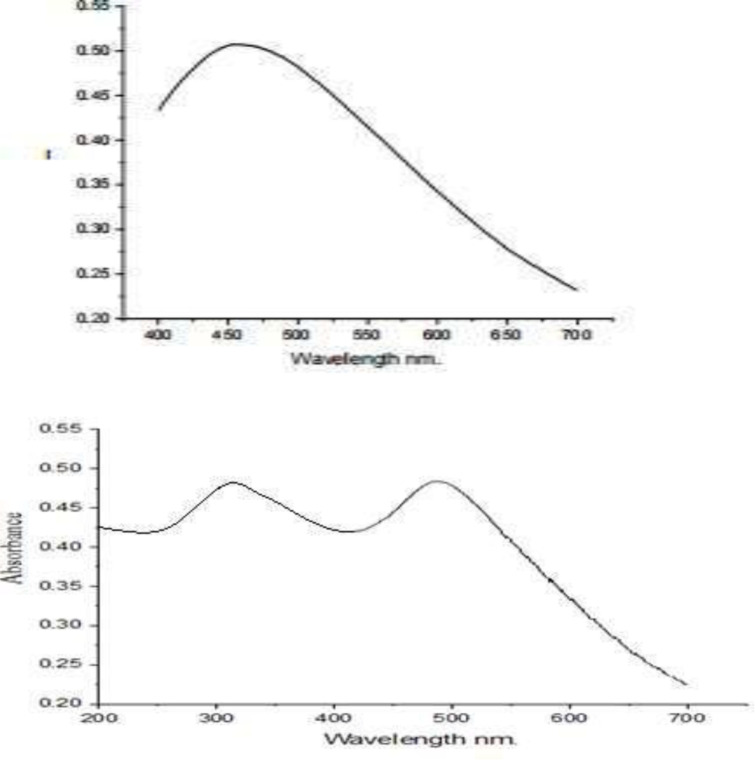
(a) Plant-Ag nanoparticles. (a) Plant-graphene nanoparticles.

Analysis of the obtained spectra showed in Fig 6a and b revealed distinct features indicative of the presence of Ag nanoparticles and graphene within the samples. In spectrum A, characteristic peaks associated with Ag nanoparticles were observed within the wavelength range of 400 to 700 nm, with a notable peak centered at 460 nm. These peaks are consistent with previous observations of Ag nanoparticles and are attributed to the surface plasmon resonance phenomenon exhibited by metallic nanoparticles. Conversely, spectrum B exhibited an additional peak around 265 nm, distinct from the Ag nanoparticle peaks. This additional peak corresponds to the presence of graphene within the sample. The appearance of this peak signifies the absorption behavior characteristic of graphene-based materials in the UV-Visible spectrum.

The disparity in the number and position of peaks between spectra A and B suggests the formation of Plant-Ag-Graphene nanocomposites from the silver nanoparticles. The emergence of an additional peak in spectrum B, indicative of graphene, alongside the Ag nanoparticle peaks, provides compelling evidence for the synthesis of these composite materials. This synthesis is further supported by the unique optical properties conferred by the combination of Ag nanoparticles and graphene within the nanocomposite structure.

The UV-Visible spectroscopy analysis thus underscores the successful integration of multiple components into the nanocomposite material, offering insights into its optical characteristics and paving the way for tailored applications in fields such as sensing, catalysis, and optoelectronics. Results were aligning with the findings of Kumar et al. [28], who observed that UV–visible spectroscopy showed surface plasmon resonance at 470 nm clearly reveals the formation of AgNPs. The results were also closely in line with the findings of Govindasamy et al. [27], who observed that the plant extract and metal NPs exhibited broad UV absorption with enhanced intensity at 288 nm.

### 3.6. Anticancer activities

**Fig 7:**
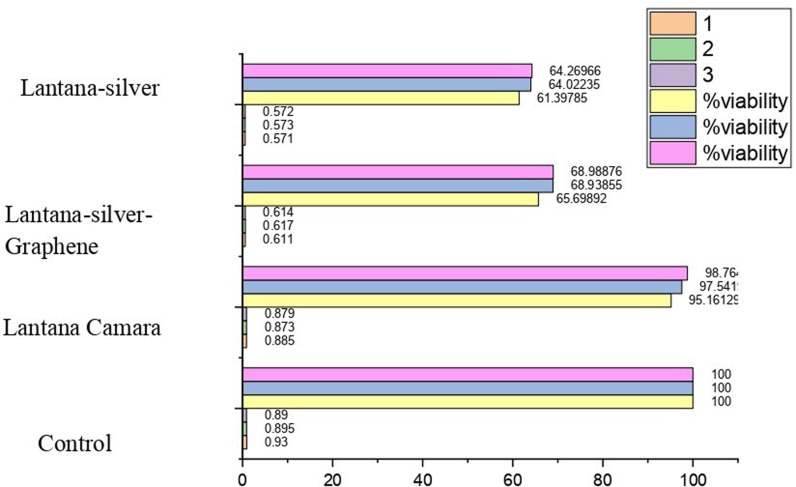
Anti-cancer activities.

The cytotoxicity assessment of graphene-silver nanocomposites and plant-catalyzed silver nanocomposites was conducted to evaluate their anti-proliferative activity. The results, summarized in the Fig 7, demonstrate a progressive enhancement in cytotoxic activity from the plant extract (*Lantana camara*) to plant-catalyzed silver nanocomposites, and further enhancement to plant-silver-graphene nanocomposites. At a concentration of 100 µg/mL, the viability of cells treated with *Lantana camara* plant extract has been found as 95.16%, however, plant-silver-graphene nanocomposite was found to be 68.98%, which was significantly higher compared to both plant-silver nanocomposites (64.26%). Subsequently, upon increasing the concentration of the compound, a consistent cell viability was maintained at 500 µg/Ml among Plant-Ag-Graphene (65.69%) and Plant-Ag (61.39%), as depicted in the Fig 7. Moreover, assuming linearity, at 50 µg/mL, the IC50 viability was 47.58% for *Lantana camara*, 32.13% for plant-silver nanocomposites and 34.49% for plant-silver-graphene nanocomposites.

These findings suggest a dose-dependent cytotoxic response, wherein higher concentrations of the plant-silver-graphene nanocomposites exhibit sustained anti-proliferative effects on the tested cells. The observed increase in cytotoxicity from plant-silver nanocomposites to plant-silver-graphene nanocomposites underscores the potential synergistic effects of incorporating graphene into the nanocomposite structure, possibly enhancing its therapeutic efficacy. Results were in line with the findings of Altabbaa et al. [29], who developed ZnO NPs using *Lantana camara* as green sources, which possess strong antibacterial properties against harmful microbes. Another study has been aligned with the findings of Mohi and Jdyea [30], who synthesized Gold nanoparticles using *Lantana camara* as a bioactive source, the particles have showed cytotoxicity against cancerous tumors through their effect on the cells of the liver cancer cell line (HEPG2) and compared it with the normal cells (WRL68) and the degree of its effect depends on the concentration. The anticancer activity of silver nanoparticles (AgNPs) was assessed, *Moringa oleifera* leaves were used as reducing and stabilizing agents to synthesize AgNPs. Results showed that the M. oleifera–AgNPs decreased the expression of CTNNB1 and LRP6 genes, while the LRP5 gene expression increased in both cell lines. The study demonstrated that herbal extracts have been excellent inhibitors of cytotoxic activities [31].

## 4. Conclusion

Various analytical techniques have extensively characterized Plant-Ag-graphene nanocomposites, offering valuable insights into their structural and chemical properties. The inclusion of graphene within silver nanoparticles was confirmed through alterations in FTIR peaks, distinct stacking patterns visible in SEM images, and EDX spectra indicating reduced silver content.

XRD analysis supported the crystalline nature of the nanocomposites, showcasing a face-centered cubic shape for Ag metal and a hexagonal structure for graphene. UV-Vis spectroscopy revealed shifts in peak positions, affirming the successful synthesis of Plant-Ag-graphene nanocomposites.

Moreover, investigations into anticancer activities demonstrated a notable escalation in cytotoxicity from plant-catalyzed silver nanocomposites to plant-silver-graphene nanocomposites, with the latter exhibiting the most potent anti-proliferative effect. This study lays the groundwork for comprehending the synthesis, characterization, and potential biomedical applications of Plant-Ag-graphene nanocomposites.
